# Structural Insights into the Interaction of Filovirus Glycoproteins with the Endosomal Receptor Niemann-Pick C1: A Computational Study

**DOI:** 10.3390/v13050913

**Published:** 2021-05-14

**Authors:** Manabu Igarashi, Takatsugu Hirokawa, Yoshihiro Takadate, Ayato Takada

**Affiliations:** 1 Division of Global Epidemiology, International Institute for Zoonosis Control, Hokkaido University, Sapporo 001-0020, Japan; ytakadate@czc.hokudai.ac.jp (Y.T.); atakada@czc.hokudai.ac.jp (A.T.); 2 International Collaboration Unit, International Institute for Zoonosis Control, Hokkaido University, Sapporo 001-0020, Japan; 3 Transborder Medical Research Center, University of Tsukuba, Tsukuba 305-8575, Japan; t-hirokawa@md.tsukuba.ac.jp; 4 Division of Biomedical Science, University of Tsukuba, Tsukuba 305-8575, Japan; 5 Cellular and Molecular Biotechnology Research Institute, National Institute of Advanced Industrial Science and Technology, Koto-ku, Tokyo 135-0064, Japan; 6 Hokudai Center for Zoonosis Control in Zambia, School of Veterinary Medicine, University of Zambia, P.O. Box 32379, Lusaka 10101, Zambia

**Keywords:** filovirus, ebolavirus, marburgvirus, glycoprotein, Niemann-Pick C1, structure, molecular modeling, molecular dynamics

## Abstract

Filoviruses, including marburgviruses and ebolaviruses, have a single transmembrane glycoprotein (GP) that facilitates their entry into cells. During entry, GP needs to be cleaved by host proteases to expose the receptor-binding site that binds to the endosomal receptor Niemann-Pick C1 (NPC1) protein. The crystal structure analysis of the cleaved GP (GPcl) of Ebola virus (EBOV) in complex with human NPC1 has demonstrated that NPC1 has two protruding loops (loops 1 and 2), which engage a hydrophobic pocket on the head of EBOV GPcl. However, the molecular interactions between NPC1 and the GPcl of other filoviruses remain unexplored. In the present study, we performed molecular modeling and molecular dynamics simulations of NPC1 complexed with GPcls of two ebolaviruses, EBOV and Sudan virus (SUDV), and one marburgvirus, Ravn virus (RAVV). Similar binding structures were observed in the GPcl–NPC1 complexes of EBOV and SUDV, which differed from that of RAVV. Specifically, in the RAVV GPcl–NPC1 complex, the tip of loop 2 was closer to the pocket edge comprising residues at positions 79–88 of GPcl; the root of loop 1 was predicted to interact with P116 and Q144 of GPcl. Furthermore, in the SUDV GPcl–NPC1 complex, the tip of loop 2 was slightly closer to the residue at position 141 than those in the EBOV and RAVV GPcl–NPC1 complexes. These structural differences may affect the size and/or shape of the receptor-binding pocket of GPcl. Our structural models could provide useful information for improving our understanding the differences in host preference among filoviruses as well as contributing to structure-based drug design.

## 1. Introduction

The genera *Marburgvirus* and *Ebolavirus* are included in the family *Filoviridae*. Filoviruses in these genera are zoonotic pathogens that often cause severe hemorrhagic fever in humans and nonhuman primates. The genus *Marburgvirus* includes a single species with two viruses: Marburg virus (MARV) and Ravn virus (RAVV), while *Ebolavirus* includes six distinct species, namely Ebola virus (EBOV), Sudan virus (SUDV), Taï forest virus (TAFV), Bundibugyo virus (BDBV), Reston virus (RESTV), and Bombali virus (BOMV) [1]. Of these, two marburgviruses (MARV and RAVV) and four ebolaviruses (EBOV, SUDV, TAFV, and BDBV) are known human-pathogenic filoviruses [2]. Moreover, EBOV is the most virulent and has caused the highest number of reported outbreaks in humans. The largest EBOV outbreak to date occurred from 2013 to 2016 in West Africa, resulting in over 28,000 cases including 11,000 deaths. Therefore, most research efforts toward the development of vaccines and therapeutics against filoviruses have largely focused on EBOV; however, there is also a need for the development of countermeasures against other filoviruses [3,4,5].

Filoviruses have a single envelope glycoprotein (GP) that is responsible for viral attachment, entry, and membrane fusion. This surface GP molecule is a homotrimer; each monomer consists of disulfide-linked subunits GP1 and GP2. GP1 contains the receptor-binding site (RBS), glycan cap, and mucin-like domain, while GP2 contains the fusion loop and transmembrane domain [6]. Following attachment of GP to cell surface attachment factors (e.g., C-type lectins), filoviruses enter cells through macropinocytosis [7,8,9]. In the late endosome, GP is cleaved by host proteases (e.g., cathepsins L and B), followed by the removal of the glycan cap and mucin-like domain [10]. The cleaved GP (GPcl), containing the exposed putative RBS, then binds to the endosomal receptor, Niemann-Pick C1 (NPC1), leading to membrane fusion [11,12].

**Figure 1:**
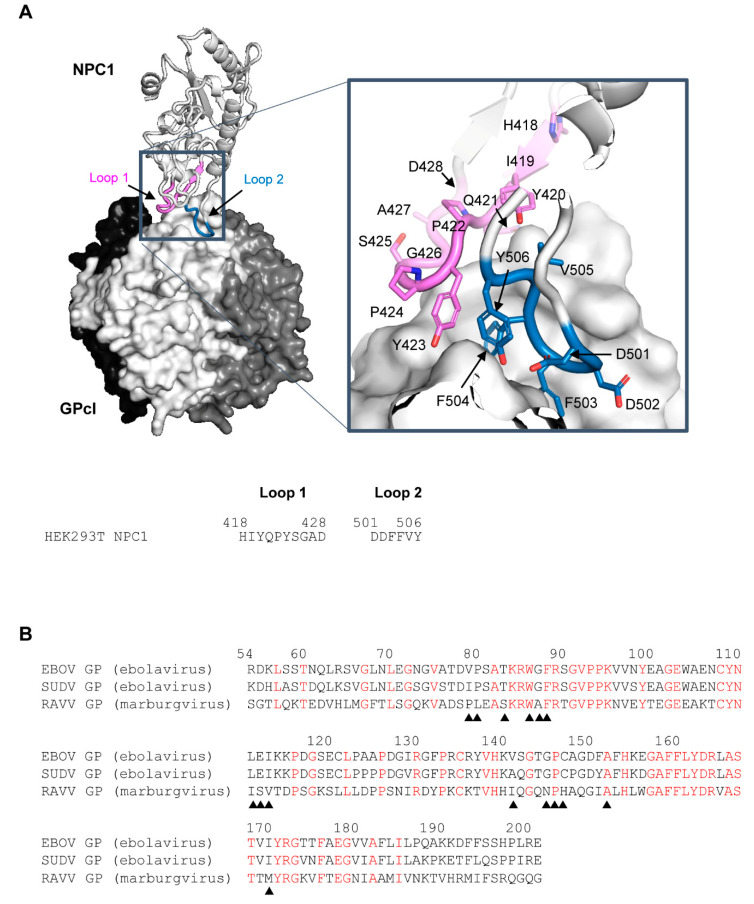
Three-dimensional structure of the EBOV GPcl–NPC1 complex and amino acid sequences of the receptor-binding domain of EBOV, SUDV, and RAVV GPs. (**A**) The three-dimensional structures of EBOV GPcl trimer and human NPC1-C (PDB ID: 5F1B) are represented as a surface and a ribbon model, respectively. On the GPcl trimer, one monomer (center) is colored white and the others are colored black and dark gray. The GPcl-binding interface, including NPC1 loop 1, and loop 2 (indicated in violet and sky blue, respectively), is shown in the boxed areas. Nitrogen and oxygen atoms are shown in blue and red, respectively. The amino acid residues of loop 1 and loop 2 in NPC1 are displayed. (**B**) Three receptor-binding domain sequences of filovirus GPcl were aligned using EBOV numbering. Conserved amino acid residues among EBOV, SUDV, and RAVV GPs are shown in red. Solid triangles indicate the positions of contact residues of EBOV GPcl with NPC1 observed in the crystal structure.

Recently, the crystal structure of EBOV GPcl in complex with human NPC1 domain C (NPC1-C) was reported [13]. The molecular interaction between EBOV GPcl and NPC1-C is mediated by two protruding loops of NPC1-C (loop 1 and loop 2), which bind to a hydrophobic pocket in RBS on the head of GPcl (Figure 1A). Computational and experimental studies based on the complex structure revealed that this pocket could be a promising target for the development of peptide-based EBOV-entry inhibitors [14]. Importantly, as both ebolaviruses and marburgviruses require GPcl binding to NPC1 to facilitate infection, the pocket serves as a target for panfilovirus inhibitors. However, the binding pocket on the head of GPcl is large, flat, and composed of hydrophobic amino acids, making it difficult to design small molecules that target the pocket of RBS [13,15]. Hence, further detailed information on the complex structure of NPC1 and GPcl is required.

EBOV GP shares approximately 60% and 30% amino acid identity with other ebolavirus and marburgvirus GPs, respectively [16,17]. However, with regard to the receptor-binding domain (residues 54–201: EBOV numbering), EBOV shares 80% and 41% sequence identity with SUDV and RAVV, respectively [6,18] (Figure 1B). Nevertheless, both ebolaviruses and marburgviruses uniformly utilize NPC1 as their essential receptor, suggesting that they are able to infect similar cell types [19]. However, recently, we found that susceptibility to filoviruses differed between certain cell lines due to unique amino acid sequences in the loops of NPC1-C, as well as a specific few amino acid differences between EBOV and MARV GPs [20]. These findings motivated us to investigate how the molecular interaction between GPcl and NPC1-C differs structurally among filoviruses. There is, however, no available information on the structure of the complex formed between NPC1 and the GPcl of filoviruses, except for EBOV.

Although the GP structures have been determined for EBOV, SUDV, BDBV, and RAVV, the available BDBV structures have low resolution [6,17,21,22]. In this study, we computationally explored the structural details of the interaction between NPC1-C and GPcl molecules of EBOV, SUDV, and RAVV via molecular modeling and molecular dynamics (MD) simulation. To clarify the factors that determine the NPC1-binding mode of GPcl, we further investigated structural differences of the NPC1-binding pocket among GPcl molecules of EBOV, SUDV, and RAVV. Our model provides structural details to support understanding of potential differences in the GPcl–NPC1 interactions among filoviruses.

## 2. Materials and Methods

### 2.1. Simulation Setup

Based on the paradigm that proteins with similar sequences and/or structures form similar complexes, we chose a template-based approach to generate initial complex structures of GPcl molecules with NPC1 [23,24,25]. Recent CAPRI experiments have demonstrated that template-based approaches generally yield more accurate predictions than free docking, provided adequate templates for the concerned complexes are available [26]. The structure of the complex formed by EBOV GPcl and NPC1-C was downloaded from the Protein Data Bank (PDB code: 5F1B). Trimer structures of SUDV and RAVV GPs, in complex with neutralizing antibodies, were also downloaded from PDB (PDB code: 3S88 and 5UQY, respectively). Based on the visible region of the EBOV GPcl–NPC1 complex (PDB code: 5F1B), the structural models of free GPcl trimers for SUDV and RAVV were then obtained from the respective GP structures by deleting the coordinates, such as the antibodies and glycan cap domains. After a single EBOV GPcl–NPC1 monomer in 5F1B was superimposed on a single monomer of SUDV or RAVV GPcl trimers, the EBOV GPcl trimer and two out of three NPC1s were removed as the GPcl trimer may simply bind to one NPC1 [27].

The free GPcl structure of EBOV was obtained from GP structures (PDB code: 6G95) by deleting the coordinates based on the visible region of GPcl in 5F1B. Free GPcl structures of SUDV and RAVV are described above. The numbering scheme for GPs was adapted from EBOV.

### 2.2. Molecular Dynamics Simulations

MD simulations were performed for the free GPcl molecules of EBOV, SUDV, and RAVV, and those complexed with NPC1-C. The AMBER ff14SB force field was used for all simulations [28]. The protonation states of the ionizable residues were assigned at pH 7.0 using the PDB2PQR web server [29]. All missing hydrogen atoms were added to the LEaP module in AMBER 16 [30]. The total charges were neutralized by the addition of either sodium or chloride counterions. The systems were then solvated in a truncated octahedral box of TIP3P water molecules with a distance of at least 10 Å around the proteins. All energy minimizations and MD simulations were performed using the pmemd.cuda program in AMBER 16, with a cut-off radius of 10 Å for the nonbonded interactions. All systems were energy-minimized in four steps: first, only hydrogen atoms; second, water and ions; third, the side chains; and finally, all atoms. Energy minimization used the steepest descent method for 500 steps followed by the conjugate gradient method for 1500 steps. After energy minimization, the system was gradually heated from 0 K to 310 K over 300 ps with harmonic restraints (with a force constant of 1.0 kcal/mol·Å^2^). Two additional rounds of MD (50 ps each at 310 K) were performed with decreasing restraint weight from 0.5 to 0.1 kcal/mol·Å^2^. Next, 150 ns of unrestrained production run was performed, and the production trajectories were collected every 10 ps. All MD simulations were performed using the NPT ensemble and the Berendsen algorithm to control the temperature and pressure [31]. The time step was 2 fs, and the SHAKE algorithm was used to constrain all bond lengths involving hydrogen atoms [32]. Long-range electrostatic interactions were treated using the particle mesh Ewald method [33]. For each GPcl–NPC1 complex system, MD simulations were performed three times using different initial velocity distributions. For each free GP system, only one simulation was conducted. Analysis of the trajectories was performed using the CPPTRAJ module of AmberTools17.

The stability of the trajectories was assessed by monitoring the root mean square deviations (RMSDs) of the backbone Cα atoms from the initial structures of the MD simulations (Figure S1). After confirming that the RMSDs in all systems reached equilibrium within 100 ns, the trajectories extracted from the last 50 ns (i.e., 100–150 ns) were used in subsequent analyses.

### 2.3. Binding Free Energy and Per-Residue Decomposition Analysis

Binding free energies and per-residue decomposition energy values were calculated using the script of the molecular mechanics/generalized Born surface area (MM/GBSA) method in AmberTools17 (MMPBSA.py), in which a single-trajectory approach was used. The conformational entropy was not considered due to the high computational cost and low prediction accuracy. The salt concentration was set to 0.20 M.

### 2.4. Representative Structure

A trajectory clustering analysis were performed to determine the representative structure with the highest population in the MD simulations for each complex system.

### 2.5. Contact Analysis

Residue–residue contacts between NPC1-C and GPcl from EBOV, SUDV, and RAVV were analyzed using the Protein Contacts in Molecular Operating Environment (MOE) software (version 2018; Chemical Computing Group, Montreal, QC, Canada). A total of 500 snapshots from each trajectory of the last 50 ns extracted every 100 ps were used for this analysis. We focused on contact residue pairs that appeared in at least 50% of the MD simulation frames.

### 2.6. Statistical Analysis

The differences in Cα−Cα distance from the tip of the NPC1 loops among EBOV, SUDV, and RAVV were compared using the Welch t-test with Bonferroni correction for multiple comparisons. These analyses were performed with the computer program R.

### 2.7. Pocket Volume Calculations

Each monomer of the free GPcl trimer was analyzed. The POVME 3.0 software was used to calculate the volumes of the NPC1-binding pocket on the GPcl [34].

## 3. Results

### 3.1. Free Energy Decomposition Analysis

#### 3.1.1. NPC1 Loops in the GPcl–NPC1 Complexes

**Figure 2:**
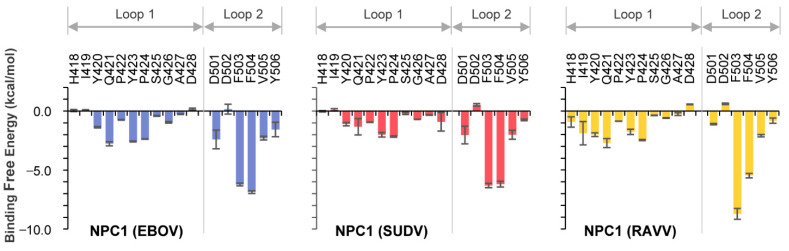
Per-residue contribution of NPC1 loops in the GPcl–NPC1 complexes to the binding free energy. Per-residue contribution to the binding free energies of loop 1 and loop 2 in the EBOV, SUDV and RAVV GPcl–NPC1 complexes were calculated using the MM/GBSA free energy decomposition method. Each MD simulation was conducted three times, and average and standard errors (SE) are shown.

The co-crystal structure of NPC1 and EBOV GPcl showed that certain amino acid residues in loop 1 and loop 2 of NPC1-C contacted the RBS on GPcl (Figure 1A). To assess which amino acid residues were involved in this interaction for EBOV, SUDV, and RAVV, we first performed MM/GBSA free energy decomposition analysis for NPC1-C (Figure 2). In each simulation, F503 and F504 located at the tip of loop 2 exhibited the highest contribution to GPcl binding. These results were consistent with the previous MD simulations of the EBOV GPcl–NPC1 complex [14]. Notably, we found that the interaction spectra for the loop 1 and 2 amino acid residues for EBOV and SUDV GPcl molecules were similar to each other; however, that of RAVV GPcl differed slightly (Figure 2). Overall, the average total binding free energies of the loops were −30.4, −26.4, and −30.9 kcal/mol for EBOV, SUDV, and RAVV, respectively, implying that SUDV GPcl had a relatively weaker interaction with NPC1 than those of EBOV and RAVV (Table 1). Moreover, the ratio of binding free energy per loop (loop 1/loop 2) was 0.37/0.63 for EBOV, 0.36/0.64 for SUDV, and 0.43/0.57 for RAVV, respectively, suggesting that NPC1 loop 2 interacts more strongly with GPcl than does loop 1. This tendency was less prominent in the NPC1–RAVV GP complex. Taken together, these results suggest that the key residues in the NPC1 loops associated with the RAVV GPcl differ from those involved in the interaction with EBOV and SUDV GPcl.

#### 3.1.2. GPcl in GPcl–NPC1 Complexes

**Figure 3:**
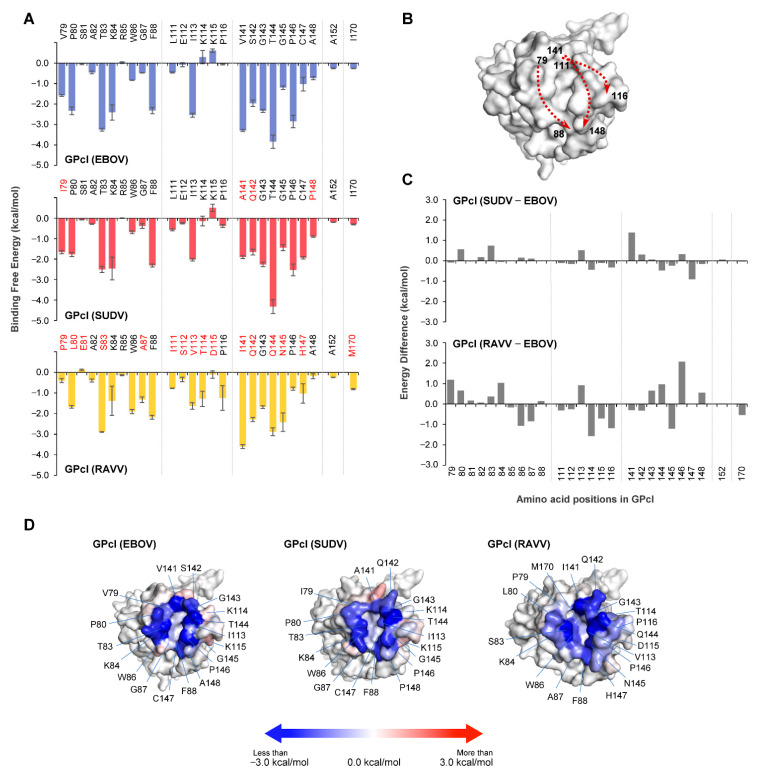
Per-residue contribution to GPcl–NPC1 binding. (**A**) Per-residue contributions to the binding free energy of GPcl in EBOV, SUDV, and RAVV GPcl–NPC1 complexes were calculated using the MM/GBSA free energy decomposition method. Amino acid residues that are distinct from those of EBOV GPcl are shown in red. The numbering scheme for GPcl was adapted from EBOV. Each MD simulation was conducted three times: data are represented as average ± SE. (**B**) The edge of the hydrophobic pocket is formed by three regions (i.e., amino acid positions 79–88, 111–116, and 141–148). Three-dimensional structures of GPcl in the EBOV GPcl–NPC1 complex are represented as surface models. Dashed arrows indicate the direction of amino acid residues, where the numbers indicate the amino acid position of the cleft of the receptor binding pocket. (**C**) Differences of binding free energies were calculated by subtraction of the value of EBOV GPcl from those of SUDV and RAVV GPcl molecules. (**D**) The three-dimensional structures of GPcl in the complexes are represented as surface models. Blue and red denote favorable and unfavorable contributions, respectively, to the binding free energy; the energies less than −3.0 kcal/mol, equal to 0 kcal/mol, and more than +3.0 kcal/mol are colored blue, white, red, respectively.

The co-crystal structure of NPC1 and EBOV GPcl shows that the hydrophobic pocket (i.e., RBS) of EBOV GPcl consists of the amino acid residues V79, P80, T83, W86, G87, F88, L111, E112, I113, V141, G145, P146, C147, A152, and I170 [13]. To identify amino acid residues important for NPC1 binding, we conducted MM/GBSA free energy decomposition analysis for the GPcl molecules of EBOV, SUDV, and RAVV (Figure 3A). Consistent with the co-crystal structure findings, the regions contributing to NPC1 binding were roughly divided into three regions (i.e., amino acid positions 79–88, 111–116, and 141–148) and two independent amino acids at positions 152 and 170. These regions formed the edges of the hydrophobic pocket (Figure 3B). In these regions, the EBOV GPcl shared 85% (22/26) and 38% (10/26) similarity with the primary sequence of SUDV GPcl and RAVV GPcl, respectively (Figure 3A). As with the results observed for the NPC1 loops, the interaction spectra of GPcl amino acid residues were comparable between the EBOV and SUDV GPcl–NPC1 complexes, but differed from that of the RAVV GPcl–NPC1 complex (Figure 3A). The comparison of per-residue binding free energy between EBOV and SUDV indicated that the amino acid residue at position 141 had an energy difference of ≥1.0 kcal/mol (Figure 3C). In contrast, seven amino acid positions (79, 84, 86, 114, 116, 145, and 146) displayed energy differences of ≥1.0 kcal/mol between EBOV and RAVV. Of these residues, the amino acid residues at positions 86, 114, 116, and 145 in RAVV GPcl had more favorable energy contributions to NPC1 binding than those in EBOV GPcl (Figure 3C). Reduced contribution of P79 and P146 to NPC1 binding also seemed to be characteristic of the RAVV GPcl–NPC1 complex. To visually understand the energy contribution of each residue, the decomposed energies were then shown on the three-dimensional structures of each GPcl molecule (Figure 3D). For example, some of the charged residues (i.e., K114 and K115) in EBOV GPcl exhibited an unfavorable contribution to NPC1 binding (Figure 3A). These residues were located outside the receptor-binding pocket. The shapes of the binding interface on GPcl were found to differ among EBOV, SUDV, and RAVV (e.g., RAVV had a wider NPC1-binding interface than EBOV and SUDV). These results suggest potential differences in the NPC1-binding modes among filoviruses.

### 3.2. Contact Analysis

**Figure 4:**
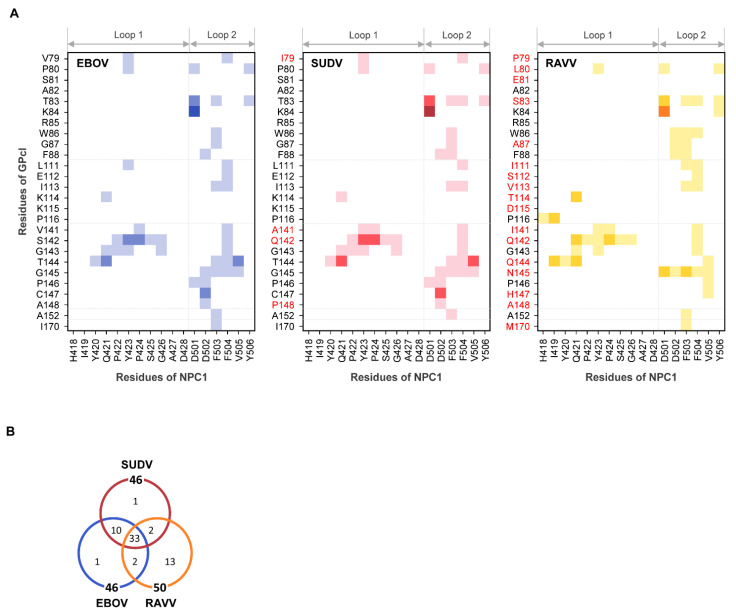
Interface contact residues in the GPcl–NPC1complexes. (**A**) Residue contact pairs of the GPcl–NPC1 interfaces for EBOV, SUDV, and RAVV were analyzed using the MOE software (version 2018; Chemical Computing Group, Montreal, Canada). Residue–residue contact pairs that appeared in at least 50% of the 1500 MD simulation frames are shown for EBOV (blue), SUDV (red), and RAVV (yellow) GPcl–NPC1 complexes. In each panel, the light, intermediate dark, and darkest colors represent the contact pairs containing van der Waals interactions (vdW), vdW + hydrogen bonds (hb), and vdW + hb + salt bridges (sb), respectively. Amino acid residues that are distinct from those of EBOV GPcl are shown in red. The numbering scheme for GPcl was adapted from EBOV. (**B**) Venn diagram for the number of residue contact pairs observed for EBOV (blue), SUDV (red), and RAVV (yellow) GPcl–NPC1 complexes.

To understand the binding modes of EBOV, SUDV, and RAVV GPcl molecules with NPC1, we examined the contact residue pairs during the MD simulations (Figure 4). There were 46, 46, and 50 residue–residue interface contacts in the EBOV GPcl–NPC1, SUDV GPcl–NPC1, and RAVV GPcl–NPC1 complexes, respectively, and 33 contact pairs were common among the viruses (Figure 4). The similarities of the contact pairs between EBOV and SUDV, EBOV and RAVV, and SUDV and RAVV were 0.88 (=43/49), 0.57 (=35/61), and 0.57 (=35/61), respectively, indicating that the contact residues were quite similar in the EBOV GPcl–NPC1 and SUDV GPcl–NPC1 complexes, but differed in the RAVV GPcl–NPC1 complex (Figure 4B). Focusing on the interaction of individual residues, we found that D501 of the NPC1 loop 2 formed hydrogen bonds and/or salt bridges with the amino acid residues at positions 83 and 84 of GPcl in all complexes (Figure 4A). F503 and F504 of loop 2 had the highest number of hydrophobic contacts to GPcl, although the contact residues of GPcl differed between EBOV, SUDV, and RAVV. These results are in agreement with the previous experimental and computational studies that imply that F503 and F504 are key residues for EBOV GPcl binding [13,14].

Additionally, RAVV GPcl was found to form unique interactions with the loops of NPC1 (Figure 4A). First, P79 in RAVV GPcl was not involved in its contact with any amino acid residues in the NPC1 loops. Second, H418 and I419 in loop 1 formed unique contacts with P116 and Q144 of RAVV GPcl. These contact sites were not observed in the EBOV and SUDV GPcl–NPC1 complexes. Third, D502 of loop 2 was in contact with amino acid residues at positions 86 and 87 of GPcl in the RAVV GPcl–NPC1 complex but not in the EBOV and SUDV GPcl–NPC1 complexes. Instead, D502 of loop 2 contacted amino acid residues at positions 145–148 of GPcl in the EBOV and SUDV GPcl–NPC1 complexes. Taken together, these results suggest that the binding structure is not considerably different between the EBOV and SUDV GPcl–NPC1 complexes; however, the RAVV GPcl–NPC1 complex has a distinct binding mode.

### 3.3. Differences in Binding Structures

**Figure 5:**
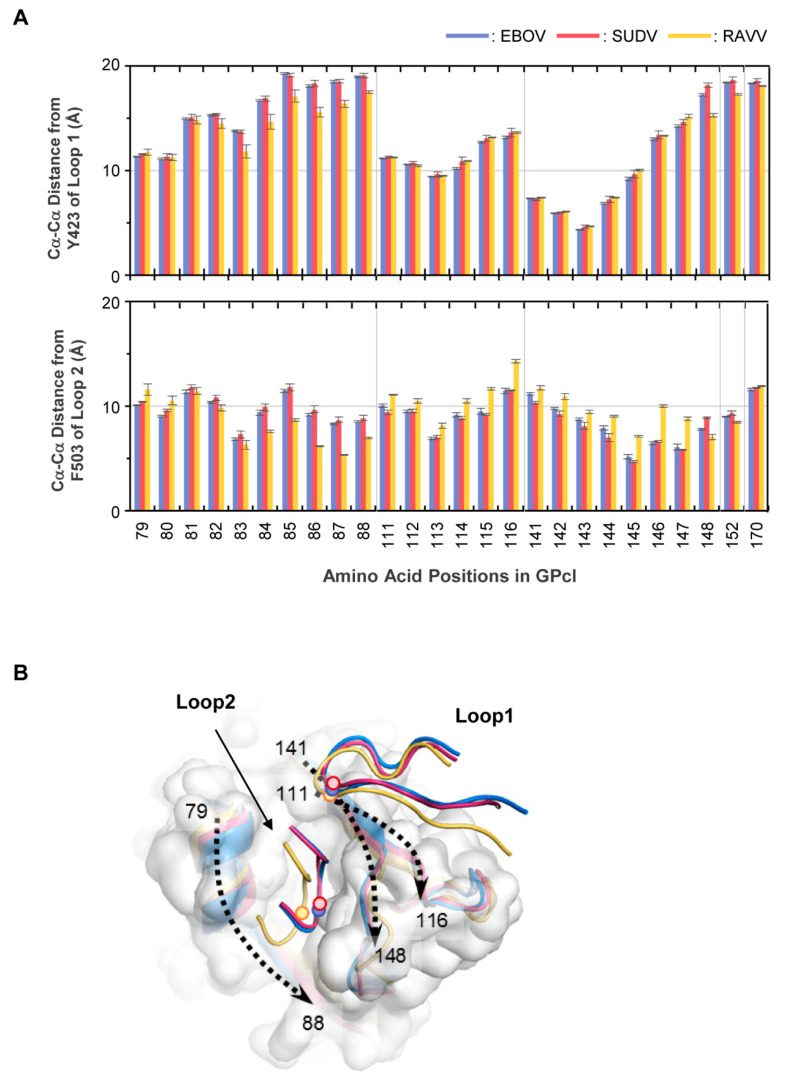
Differences in the GPcl–NPC1 binding structures between EBOV, SUDV, and RAVV. (**A**) Residue contact pairs of the GPcl–NPC1 interfaces for EBOV, SUDV, and RAVV were analyzed using the MOE software (version 2018; Chemical Computing Group, Montreal, Canada). Residue–residue contact pairs that appeared in at least 50% of the 1500 MD simulation frames, are shown for EBOV (blue), SUDV (red), and RAVV (yellow) GPcl–NPC1 complexes. In each panel, the light, intermediate dark, and darkest colors represent the contact pairs containing van der Waals interactions (vdW), vdW + hydrogen bonds (hb), and vdW + hb + salt bridges (sb), respectively. Amino acid residues that are distinct from those of EBOV GPcl are shown in red. The numbering scheme for GPcl was adapted from EBOV. (**B**) Venn diagram for the number of residue contact pairs observed for EBOV (blue), SUDV (red), and RAVV (yellow) GPcl–NPC1 complexes.

To further investigate the difference in the binding structures, we measured the Cα−Cα distances from the tip of the NPC1 loops (i.e., Y423 in loop 1 and F503 in loop 2) to the respective amino acid residues interacting with the loops (Figure 5A, Table S1). The tip of loop 1 was similarly close to the residues at positions 141−144 in the EBOV, SUDV, and RAVV GPcl–NPC1 complexes, while the distances to some of the residues were lower in the RAVV GPcl–NPC1 complex. In contrast, more notable differences were observed in the distances from the tip of NPC1 loop 2 to certain residues between EBOV/SUDV and RAVV GPcl–NPC1 complexes. The distances to the amino acid residues at positions 84−88 were significantly shorter in the RAVV GPcl–NPC1 complex than in the EBOV and/or SUDV GPcl–NPC1 complexes, and the loop 2 tip was closer to the amino acid residues at positions 114−116 and 144−147 in the EBOV and SUDV GPcl–NPC1 complexes than in the RAVV GPcl–NPC1 complex. It was also noted that the distances from the tip of loop 2 to the amino acid residues at positions 141 and 148 were significantly shorter and longer, respectively, in the SUDV GPcl–NPC1 complex than in the EBOV GPcl–NPC1 complex. The representative structures of the GPcl–NPC1 complexes clearly showed that the spatial location of the loop 2 tip was shifted toward one side of the GPcl pocket (i.e., amino acid residues at positions 79–88) in the RAVV GPcl–NPC1 complex, compared to EBOV and SUDV GPcl–NPC1 complexes (Figure 5B). In the representative structure of the SUDV GPcl–NPC1 complex, the tip of NPC1 loop 2 was slightly shifted compared to that of the EBOV GPcl–NPC1 complex, consistent with the results of Cα−Cα distance from the tip to the amino acid positions at 141 and 148.

### 3.4. Molecular Mechanism Underlying the Difference in Binding Modes for NPC1

**Figure 6:**
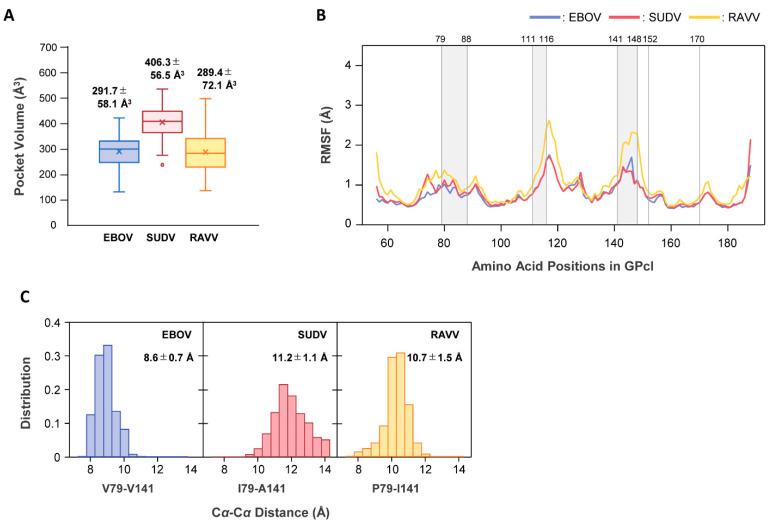
MD simulations for free GPcl molecules of EBOV, SUDV, and RAVV. (**A**) The volumes of the receptor-binding pocket in the free GPcl molecules of EBOV (blue), SUDV (red), and RAVV (yellow) were calculated with the POVME 3.0 program. The values in the plot area represent the mean and standard deviation (SD) of the pocket volumes from each monomer in the GPcl trimer. In each box plot, the mean and median values are indicated by “x” and a solid horizontal bar, respectively. The top and bottom edges of the box mark the first and third quartile, respectively. Outliers, shown as open circles, are cases with values more than 1.5 times the interquartile range away from the upper or lower quartile. The whiskers extending from the box indicate the highest and lowest values, excluding the outliers. (**B**) RMSF values were plotted for free EBOV (blue), SUDV (red), and RAVV (orange) GPcl structures. Gray regions between the numbers above the plot area represent the constituent residues of the receptor binding pocket. (**C**) The Cα−Cα distances (Å) of V79–V141, I79–A141, and P79–I141 were measured for each monomer in the GPcl trimers of EBOV, SUDV, and RAVV, respectively. Distributions of the Cα distances during MD simulations are shown for EBOV (blue), SUDV (red), and RAVV (orange) GPcl molecules. The average distance and SD are indicated in each panel. A total of 1500 snapshots from each trajectory of the last 50 ns were used for all the analyses (**A**–**C**).

To elucidate the molecular mechanism underlying the differences in binding modes for NPC1, we performed MD simulations for free GPcl of EBOV, SUDV, and RAVV (Figure 6). First, we postulated that there might be structural differences in the NPC1-binding pocket of free GPcl between EBOV, SUDV, and RAVV. The average sizes of the NPC1-binding pockets of EBOV, SUDV, and RAVV GPcl were estimated to be 291.7 Å^3^, 406.3 Å^3^, and 289.4 Å^3^, respectively, indicating that SUDV had the largest binding pocket, while those of EBOV and RAVV GPcl were comparable (Figure 6A).

To investigate the dynamic features of the binding pocket, we then calculated the root mean square fluctuation (RMSF) values, which allowed us to verify the molecular flexibility of the pocket structures (Figure 6B). Per-residue fluctuations did not show any noticeable differences between EBOV and SUDV. However, larger fluctuations were observed around amino acid residues at positions 110−122 and 136−152 in RAVV GPcl compared to EBOV and SUDV GPcl molecules.

Finally, we attempted to determine why the pocket size of SUDV was larger than those of the others. As described above, the distance from the loop 2 tip to the amino acid residues at positions 141 of GPcl was shorter in the SUDV GPcl–NPC1 complex than in the EBOV GPcl–NPC1 complex. At position 141, EBOV, SUDV, and RAVV GPcl molecules had different amino acid residues: V, A, and I, respectively (Figure 1B). Moreover, three-dimensional structures of GPcl showed that position 141 is spatially near position 79, while the amino acid residues at these positions formed part of a raised rim in the receptor-binding pocket (Figure 3B). Therefore, we measured the Cα−Cα distances between the amino acid residues at positions 79 and 141 during MD simulations (Figure 6C). We found that distributions of the distances observed in the MD simulations varied among EBOV, SUDV, and RAVV. The average distances between EBOV and SUDV, EBOV and RAVV, and SUDV and RAVV were all significantly different, and SUDV had the longest distance, supporting the possibility of an expanded size and/or shape of the receptor-binding pocket.

## 4. Discussion

In this study, molecular modeling and MD simulations were performed for EBOV, SUDV, and RAVV GPcl–NPC1 complexes, the structural details of which had not been previously elucidated. The binding structure of NPC1 and EBOV GPcl has provided important information for understanding the potential differences in host preference among filoviruses, as well as for structure-based drug design [13,14,20,35]. There is, however, no available information to date on the structure of the GPcl–NPC1 complex for other filoviruses. In the present study, we focused on SUDV and RAVV as well as EBOV and revealed that the GPcl–NPC1 binding structures of EBOV and SUDV were generally similar. However, these structures differed from that of RAVV. We first confirmed that F503 and F504 of the NPC1 loop 2 are important for GPcl binding to GPcl molecules of SUDV and RAVV as well as EBOV, although the key residues in the NPC1 loops of the RAVV GPcl–NPC1 complex differed from those in the EBOV and SUDV GPcl–NPC1 complexes. For all viruses tested, it was also noted that loop 2 has a greater contribution to NPC1 binding than loop 1.

We also observed that the differences in binding mode might be due to the structural differences in the NPC1-binding pocket between EBOV, SUDV, and RAVV GPcl molecules (Figure 6). Although the average pocket sizes of EBOV and RAVV are comparable, they differ from that of SUDV, with the SUDV GPcl exhibiting a longer distance between residues 79 and 141 than EBOV GPcl (Figure 6A,C). This is consistent with the results of the average total binding free energies of the loops (Table 1). The larger size of the NPC1-binding pocket of SUDV GPcl may cause looser and weaker binding affinity to NPC1 loops than EBOV and RAVV GPcl molecules, but it may have the potential to overcome the structural mismatch at the GPcl–NPC1 binding interface [36]. For example, a single mutation V141A in EBOV GP increased its binding affinity for NPC1 in a bat species (*Eidolon helvum*), suggesting that the V141A mutation likely creates a more sterically favorable (open) NPC1-binding site [36]. In SUDV GPcl, I79 and A141 appear to be primarily involved in the larger size of the NPC1-binding pocket (Figure 3D and Figure 6C).

Although per-residue fluctuations were not markedly different between EBOV and SUDV, larger fluctuations were observed in RAVV GPcl (Figure 6B). Both EBOV and SUDV GPcl molecules have disulfide (S–S) bonds between C121 and C147, while RAVV does not [21]. The lack of S–S bonds is likely the reason for the larger fluctuation around these amino acid positions in RAVV GPcl, which may affect the binding pocket volume (Figure 6A). In the RAVV GPcl–NPC1 complex, the root residues of NPC1 loop 1 (i.e., H418 and I419) were predicted to contact P116 and Q144 of RAVV GPcl, whereas these contact sites were not observed in the EBOV and SUDV complexes (Figure 4A). Since P116 and Q144 of RAVV GPcl exhibit large thermal fluctuation (Figure 6B), these contact sites might also be generated in response to the lack of S–S bonding. Taken together, our data suggest that the differences in binding mode between EBOV/SUDV and RAVV are caused by their pocket volume and/or the shape of the RBS on the GPcl due to the lack of S–S bonds and/or the difference in the amino acid residues forming the edge of the receptor-binding pocket.

Our structural model shows that the GPcl–NPC1 binding structure of RAVV is distinct from that of EBOV (Figure 4A and Figure 5B). We previously demonstrated that the amino acid residues in the loop 1 region are critical determinants for the differential host specificity between EBOV and MARV, in which the constituent residues of the receptor-binding pocket are 100% identical to those of RAVV [19,20]. Loop 1 of a bat-derived cell line, FBKT1, which is susceptible to EBOV, not MARV, has a unique amino acid residue E426, whereas most of the other bat and primate cell lines tested have G426. A site-directed mutagenesis study further demonstrated that cells expressing NPC1 with E426 were susceptible to EBOV GP-mediated infection, while exhibiting lower susceptibility to MARV GP-mediated infection [20]. In the present study, our contact analysis revealed that the G426 of NPC1 loop 1 directly contacted the S142 of EBOV GPcl or Q142 of RAVV GPcl (Figure 4A). Thus, it is highly likely that E426 has impaired interactions with Q142 of MARV GPcl. Accordingly, the amino substitution at position 142 (Q142S) resulted in increased infectivity of vesicular stomatitis virus (VSV) pseudotyped with MARV GP (VSV-MARV) in FBKT1 cells [20]. It was also noted that the amino acid substitution at position 87 (A87G), locating opposite the Q142 in the receptor-binding pocket, increased the sensitivity of FBKT1 cells to VSV-MARV [20]. Interestingly, A87 of RAVV GPcl is the closest residue to the tip of loop 2 (Figure 5A), suggesting that the A87G mutation of MARV GPcl likely creates an NPC1-binding site that is sterically favorable for FBKT1 NPC1.

Due to its remarkable pathogenicity and the highest number of human outbreaks, research efforts toward the development of vaccines and therapeutics against filovirus diseases have been almost exclusively focused on EBOV. Although previous computational and experimental studies have shown that the receptor-binding pocket of EBOV is a promising target for peptide-based drugs [14], information on other filoviruses is quite limited. In the present study, we predicted the three-dimensional structures of SUDV and RAVV GPcl–NPC1 complexes using in silico modeling based on the previously resolved EBOV GPcl–NPC1 structure. The receptor-binding pocket of GP is not accessible unless its glycan cap and mucin-like domain are removed, whereas it may be feasible to design inhibitors targeting the crest of the RBS. Indeed, a monoclonal antibody that binds to this crest has been shown to inhibit NPC1 binding and prevent EBOV infection [37].

In this study, we used a template-based approach to generate the initial structure of GPcl–NPC1 complexes for MD simulations. In order to assess the validity of the initial model, we performed a redocking experiment using a known crystal structure of the EBOV GPcl–NPC1 complex (PDB ID: 5F1B). Even though this structure did not have any apparent steric hindrances or other unfavorable interactions, the redocking study using three sophisticated tools (MOE, ZDOCK [38], and ClusPro [39]) did not generate the near-native structure (i.e., near-crystal structure). Protein–protein docking simulations sometimes fail to reveal the correct structures. In particular, it has been reported that when the interaction area is small (ΔASA < 1400 Å^2^), protein–protein docking is very difficult [40]. For the EBOV GPcl–NPC1 complex, the calculated area was approximately 1395 Å^2^. When redocking of a crystal structure complex fails in this way, the docking of similar targets is also usually unsuccessful. In fact, our initial structure models of the SUDV GPcl–NPC1 and RAVV GPcl–NPC1 complexes were not reproducible by docking experiments, just like the crystal structure of the EBOV GPcl–NPC1 complex. However, because a good template (i.e., 5F1B) was available in this study, it is probably more reliable to generate initial structures of GPcl–NPC1 complexes for MD simulations via the template-based approach than by free docking [26]. Moreover, the RMSD values at equilibrium state of the three independent MD simulations in each complex were similar (Figure S1). A ligand-free, flexible biomolecule in solution attains the global minimum energy conformation after ligand binding [41,42]. The RMSD values might show this phenomenon, supporting the possibility that the initial structure models used in this study were reasonable. Although the present computational study must be supported by experimental data, our approach may provide new perspectives on the understanding of the host cell tropism of filoviruses, as well as the structure-based drug design of filovirus entry inhibitors.

## Figures and Tables

**Table 1:** Estimated average total binding free energies between NPC1 loop and GPcl of EBOV, SUDV, and RAVV.

	EBOV	SUDV	RAVV
Loop 1	−11.2 ± 0.3 (0.37)	−9.6 ± 0.4 (0.36)	−13.3 ± 1.3 (0.43)
Loop 2	−19.2 ± 0.6 (0.63)	−16.8 ± 1.1 (0.64)	−17.6 ± 0.0 (0.57)
Total	−30.4 ± 0.4 (1.00)	−26.4 ± 0.9 (1.00)	−30.9 ± 1.3 (1.00)

Average total binding free energies (in kcal/mol) were calculated by adding the energies of each residue of the loops. Data are shown as the mean ± standard error (SE) of three MD simulations. The values in parentheses represent the ratios of loop 1 and loop 2.

## Supplementary Materials

The following are available online at https://www.mdpi.com/article/10.3390/v13050913/s1, Figure S1: Root mean square deviation (RMSD) of the EBOV, SUDV, and RAVV GPcl–NPC1 complexes; Table S1: *p* Values for the multiple comparisons of the distance from the NPC1 loops to each residue of GPcl.
