# Supplementary material for: Structural Insights into the Interaction of Filovirus Glycoproteins with the Endosomal Receptor Niemann-Pick C1: A Computational Study
Source: Viruses. 2021 May 14;13(5):913. doi: 10.3390/v13050913 (PMC8156010; doi:10.3390/v13050913)
Supplement: Supplementary file 1 [file viruses-13-00913-s001.zip › viruses-1170959-supplementary.pdf]

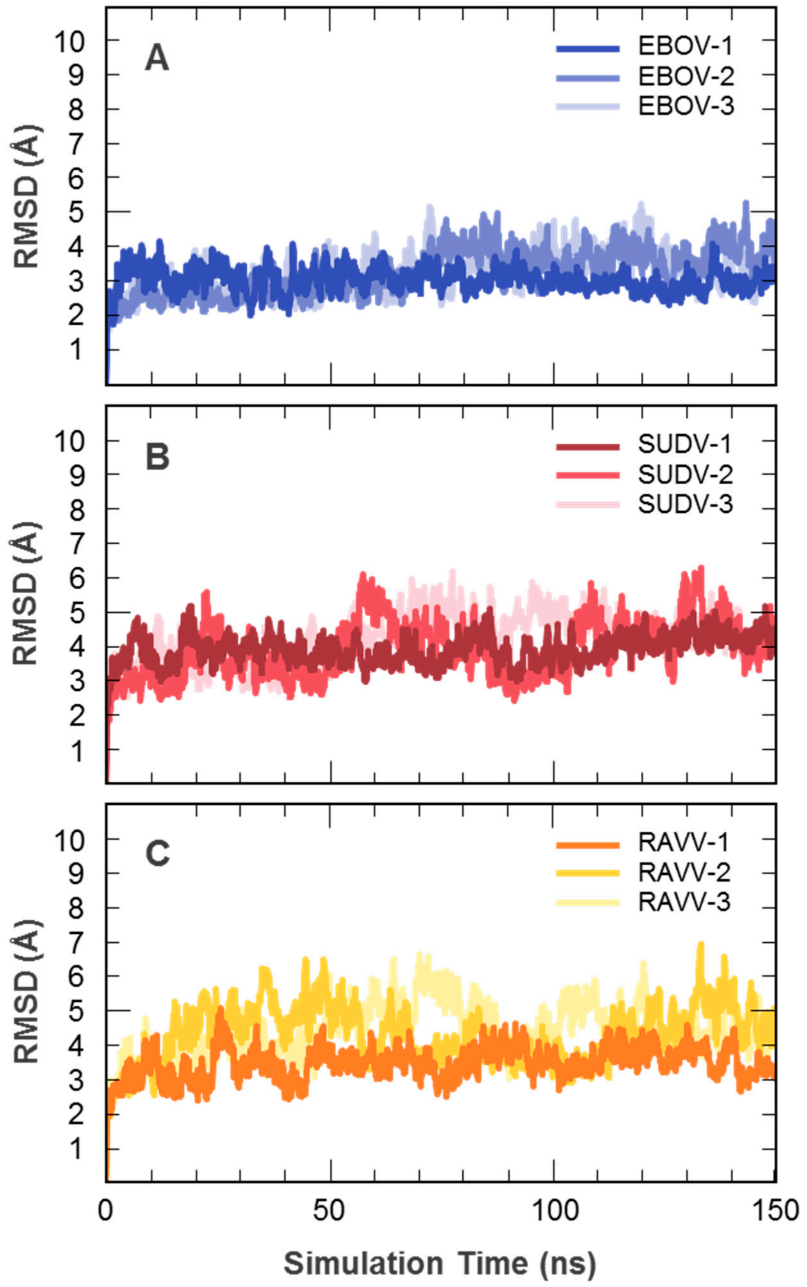

Figure S1. Root mean square deviation (RMSD) of the EBOV, SUDV, and RAVV GPcl-NPC1 complexes

Table S1. P-values for the multiple comparisons of the distance from the NPC1 loops to each residue of GPcl (EBOV vs SUDV, EBOV vs RAVV, and SUDV vs RAVV)

	Loop1			Loop 2		
residue number	EBOV vs SUDV	EBOV vs RAVV	SUDV vs RAVV	EBOV vs SUDV	EBOV vs RAVV	SUDV vs RAVV
79	0.034	0.298	0.523	0.038	0.107	0.157
80	0.557	0.721	0.842	0.022	0.068	0.158
81	0.600	0.799	0.582	0.148	0.796	0.414
82	0.517	0.223	0.189	0.251	0.220	0.081
83	0.736	0.078	0.079	0.301	0.296	0.126
84	0.543	0.097	0.072	0.329	0.007	0.018
85	0.440	0.061	0.065	0.422	0.000	0.004
86	0.499	0.023	0.008	0.266	0.000	0.010
87	0.960	0.012	0.009	0.269	0.000	0.004
88	0.740	0.002	0.010	0.292	0.000	0.008
111	0.202	0.375	0.409	0.077	0.017	0.011
112	0.282	0.455	0.172	0.856	0.020	0.021
113	0.448	0.352	0.618	0.721	0.019	0.020
114	0.116	0.011	0.861	0.276	0.010	0.004
115	0.380	0.009	0.782	0.359	0.009	0.000
116	0.385	0.028	0.994	0.871	0.001	0.004
141	0.369	0.188	0.249	0.009	0.062	0.005
142	0.221	0.022	0.460	0.147	0.049	0.014
143	0.238	0.002	0.878	0.230	0.018	0.052
144	0.336	0.009	0.637	0.133	0.015	0.030
145	0.325	0.012	0.403	0.128	0.004	0.000
146	0.388	0.152	0.838	0.589	0.000	0.000
147	0.247	0.015	0.162	0.498	0.002	0.002
148	0.048	0.002	0.001	0.003	0.085	0.009
152	0.305	0.003	0.017	0.171	0.006	0.035
170	0.154	0.004	0.060	0.223	0.055	0.048

Using Bonferroni correction, P values of less than 0.017 (0.05/3) were considered statistically significant (pink).
